# Schistosomes and snails: a molecular encounter

**DOI:** 10.3389/fgene.2014.00230

**Published:** 2014-07-21

**Authors:** Matty Knight, Halime D. Arican-Goktas, Wannaporn Ittiprasert, Edwin C. Odoemelam, André N. Miller, Joanna M. Bridger

**Affiliations:** ^1^Department of Microbiology, Immunology and Tropical Medicine, The George Washington UniversityWashington, DC, USA; ^2^Biosciences, Health Sciences and Social Care, Brunel University LondonLondon, UK; ^3^Schistosomiasis, Biomedical Research InstituteRockville, MD, USA

**Keywords:** intermediate snail host, *B. glabrata*, *S. mansoni*, resistance, susceptibility, compatibility, gene-expression, gene loci re-localization

## Abstract

*Biomphalaria glabrata* snails play an integral role in the transmission of *Schistosoma mansoni*, the causative agent for human schistosomiasis in the Western hemisphere. For the past two decades, tremendous advances have been made in research aimed at elucidating the molecular basis of the snail/parasite interaction. The growing concern that there is no vaccine to prevent schistosomiasis and only one effective drug in existence provides the impetus to develop new control strategies based on eliminating schistosomes at the snail-stage of the life cycle. To elucidate why a given snail is not always compatible to each and every schistosome it encounters, *B. glabrata* that are either resistant or susceptible to a given strain of *S. mansoni* have been employed to track molecular mechanisms governing the snail/schistosome relationship. With such snails, genetic markers for resistance and susceptibility were identified. Additionally, differential gene expression studies have led to the identification of genes that underlie these phenotypes. Lately, the role of schistosomes in mediating non-random relocation of gene loci has been identified for the first time, making *B. glabrata* a model organism where chromatin regulation by changes in nuclear architecture, known as spatial epigenetics, orchestrated by a major human parasite can now be investigated. This review will highlight the progress that has been made in using molecular approaches to describe snail/schistosome compatibility issues. Uncovering the signaling networks triggered by schistosomes that provide the impulse to turn genes on and off in the snail host, thereby controlling the outcome of infection, could also yield new insights into anti-parasite mechanism(s) that operate in the human host as well.

## INTRODUCTION

The neglected tropical disease (NTD), schistosomiasis, ranks second only to malaria as one of the most persistent debilitating diseases in impoverished areas of the tropics and sub-tropics, mainly in sub-Saharan Africa (Jenkins-Holick and Kaul, 2013). Freshwater snails are the obligate intermediate hosts for the transmission of the causative schistosome parasite. Using a combination of mass drug administration (MDA), mainly in school-aged children, and molluscicides to eliminate the snail from fresh water bodies, such as lakes, rivers, and their tributaries, low prevalence of schistosomiasis has now been achieved in several endemic countries (Tchuem Tchuente et al., 2013; Olveda et al., 2014). Despite these control efforts, however, reduction of schistosomiasis for the long term remains elusive, especially in poorer countries where access to clean water and sanitation remains challenging. Increasingly, strong advocacy by organizations, such as the schistosomiasis control initiative (SCI) and schistosomiasis consortium organization research effort (SCORE) have called for a renewed effort to better detect the parasite in snail populations where low prevalence of schistosomiasis has been accomplished (Fenwick et al., 2009a,b; Garba et al., 2009; Musuva et al., 2014). It is believed that by using an integrated control approach with aggressive surveillance complete global elimination of schistosomiasis might even become possible (Rollinson et al., 2013).

To make this goal a reality, however, novel intervention tools targeting the intermediate snail host are needed, especially since only one drug, praziquantel, effectively treats schistosomiasis and no vaccine is available (Hotez et al., 2010; Chai, 2013). Left untreated, schistosome adult worms can survive for several years (between 5 and 30 has been recorded). Eggs from mating not passed with human excreta but remaining trapped in tissues are the main cause of the pathology associated with schistosomiasis. This depends on the infecting parasite species, for example *Schistosoma haematobium,* can in time cause bladder cancer expedited from eggs sitting in tissues of this organ. Most notably, chronic *S. haematobium* infection can also cause female genital schitosomiasis (FGS), a condition that results in infertility and predisposes this section of society to HIV infection (Kjetland et al., 2010; Downs et al., 2013; Owusu-Bempah et al., 2013; Sahu et al., 2013).

Given the current interest in eliminating schistosomiasis, frequent use of molluscicides can have a negative impact on the environment as well as damage delicate ecosystems. Therefore, as was suggested as early as in the 1950’s, a form of biological control can be adopted by using incompatible snails to replace resident susceptible ones in endemic foci (Ruiz-Tiben et al., 1969; Ferguson and Ruiz-Tiben, 1971; Jobin and Laracuente, 1979). Indeed, in a proof of concept study conducted in the Caribbean Island of St Lucia, the snail *Biomphalaria straminae* a secondary host of the parasite *S. mansoni*, not its compatible snail *B. glabrata,* was utilized to eliminate schistosomiasis in the part of the island where this form of control was adopted (Pointier, 1993). In a more recent Brazilian study, the introduction of parasite resistant strains of *B. tenagophila* into an endemic site was found to reduce transmission as cross hybridization between resident susceptible and introduced snails increased over time (De Almeida Marques et al., 2014). This alternative intervention method, focusing on reducing schistosomiasis by blocking the snail stage of the parasite life cycle although attractive, will require a better understanding of the molecular basis of the snail and parasite interaction. This review will highlight recent advances that have been made toward unraveling molecular mechanisms that underlie parasite development in the snail host, particularly in the role of spatial epigenetics in the outcome of schistosome infection in the snail host.

## GENETICS OF THE SNAIL HOST AND HOW THE PARASITE SHAPES THE OUTCOME OF INFECTION

Over the past two decades a major shift occurred in studies investigating snail/schistosome compatibility issues, from a comparative immunological point of view to one with a more molecular focus. Facilitating these studies was the existence of resources enabling investigators to identify genes involved in the snail’s behavior toward the parasite. These include the *B. glabrata* embryonic cell line (Bge), and snail stocks that have been bred to display either resistance or susceptibility to *S. mansoni*.

Susceptibility of the snail host to the parasite has a genetic basis with genes of both the snail and parasite affecting the outcome (Spada et al., 2002; Theron and Coustau, 2005; Oliveira et al., 2008). For instance, resistance to infection in adult snail stocks, such as BS-90, 13-16-R1, and 10-R2 is a single dominant gene trait that is heritable by simple Mendelian genetics (Knight et al., 1991; Spada et al., 2002). Resistance in juvenile snails, however, is a complex trait that is controlled by several genes, five or six, each with multiple alleles (Ittiprasert et al., 2010).

Susceptibility to *S. mansoni* in *B. glabrata* snail stocks, such as M-line and NMRI is also a complex trait either as adults or juveniles. In some cases, snails can display susceptibility as juveniles, become resistant as adults, and revert to the susceptibility phenotype once they age (Knight et al., 1991; Richards et al., 1992). All these different outcomes of the parasite/snail encounter points to a relationship that is more variable and unpredictable in the snail than in the human host. Indeed, while a given parasite strain can only develop within a compatible snail, no such discrimination occurs in the human host. For this reason, it is highly plausible that discovery, in the snail host, of molecular pathways that either prevent or facilitate parasite development, if evolutionarily conserved, will help in designing tools that can be utilized for targeted control of schistosomiasis in both the snail vector and the human host.

## GENE DISCOVERY HAS BEEN ACCOMPLISHED BY EXAMINING DIFFERENTIAL GENE EXPRESSION IN RESISTANT, AND SUSCEPTIBLE SNAILS

To determine the effect of parasite infection on *B. glabrata* gene expression, snail stocks that are either resistant (BS-90) or susceptible (NMRI, and M-line), exposed at various time points to *S. mansoni*, were examined by a variety of approaches to assess the degree of parasite-mediated modulation of the snail transcriptional profile. The variety of methods utilized to examine differential gene expression between these snails, included generating expressed sequence tags (ESTs) from either intact snails or specific tissues (hemocytes, ovotestis, mantle, hepatopancreas, and albumen gland), differential display reverse transcription-polymerase chain reaction (DD-RT-PCR), microarray analysis, and RNA-sequencing (RNA-seq; Knight et al., 2000; Raghavan et al., 2003; Mitta et al., 2005; Lockyer et al., 2007, 2008; Ittiprasert et al., 2009, 2010). In addition, a suppression subtractive hybridization (SSH)-cDNA cloning strategy was employed to enrich for transcripts featuring prominently between resistant and susceptible snails during the early response phase to the parasite (Ittiprasert et al., 2010).

From these various profiling experiments, results showed that transcripts involved in the snail’s innate defense system (IDS) were amongst those that were most significantly upregulated after early exposure to the parasite, especially in resistant snails. Thus in resistant snails, such as the BS-90 stock, compared to susceptible ones, e.g., the NMRI, and M-line snail stocks, immune-defense behavior of the snail/schistosome interplay, involving cellular, “macrophage-like” cells known as hemocytes, and the plasma, hemolymph, component, recognize the incoming parasite (larval miracidium) as non-self by an as yet unknown mechanism. This recognition results in the encapsulation of the parasite by hemocytes released into the open circulatory system from the snail’s hemopoietic tissue, the amoebocyte producing organ (APO) located in the pericardiac and renal regions (Souza Sdos and Andrade, 2006, 2012). The nature of the excretory-secretory products (ESPs) released from the parasite that stimulate hemocyte production, recruitment, and attachment to the parasite surface, remain unknown. However, hemocyte genes that are up-regulated in response to exposure to schistosomes and their ESPs are being identified (Zahoor et al., 2009, 2014; Lockyer et al., 2012). Aided by plasma products, including lectins, hemocytes encapsulate the parasite within a few hours post-exposure, and by a cytotoxic mechanism(s) that include the production of reactive oxygen and nitrogen intermediates, kill the parasite (Hahn et al., 2001).

Hemocytes passively transferred from a resistant to susceptible snail have been shown to be capable of rendering these snails less susceptible (Vasquez and Sullivan, 2001). This active anti-schistosome defense reaction in resistant snails does not occur in those that are compatible/susceptible. In this case, the prevailing hypothesis is that the parasite is capable of suppressing the snail IDS, leading to the successful establishment and development of the early mother sporocyst stage (Yoshino and Bayne, 1983; Mattos et al., 2011). What mechanism(s) are involved in the manipulation of the snail’s defense by the parasite has yet to be uncovered but from comprehensive transcriptomics, several lectins, including highly diversified transcripts encoding the fibrinogen encoding proteins, collectively known by the acronym fibrinogen-related proteins (FREPs) are upregulated in both resistant and susceptible snails. Studies using experimental dsRNA to knock down the transcript for FREP 3 in resistant snails rendered them susceptible, indicating that FREPs plays a role in anti-trematode defense not coagulation (Zhang and Loker, 2004; Hanington et al., 2012). The unusual diversification of FREPs, the first to be discovered for a defense-related molecule in invertebrates, points to an IDS that is more complex than was previously envisioned. Snail lectins, including FREPs also bind to released parasite molecules, such as mucin (Roger et al., 2008a; Zhang et al., 2008; Hanington et al., 2010). This interaction between snail and parasite molecules that have co-evolved sufficiently to “match” has been proposed as underlying snail/schistosome compatibility (Massoud et al., 2004; Oliveira et al., 2008; Roger et al., 2008a,b; Mitta et al., 2012). Lately, evidence for specific-genotype immune priming has also been detected in the *B. glabrata*/schistosome interaction (Portela et al., 2013). Thus far, however, interacting components known to trigger innate immunity in other invertebrates, specifically pathogen-associated molecular patterns (PAMPs), as well as host pattern recognition receptors (PRRs), including signaling PRRs, e.g., the membrane-bound Toll receptors remain to be characterized for the *B. glabrata/S. mansoni* relationship (Meurs et al., 2011; Paveley et al., 2011).

Considerable progress is being made on identifying shared molecular moieties in the parasite and snail host, and evidence is emerging that shows the existence of shared specific glycotypes (glycan epitopes) between the larval schistosome parasite and snail hemocytes (Yoshino et al., 2013). It is believed that the shared epitopes, also called molecular mimicry might be used to dampen immune recognition of the parasite thus evading immune detection.

## STRESS INDUCTION BY SCHISTOSOMES IN THE SNAIL HOST CORRELATES WITH SUSCEPTIBILITY

**FIGURE 1:**
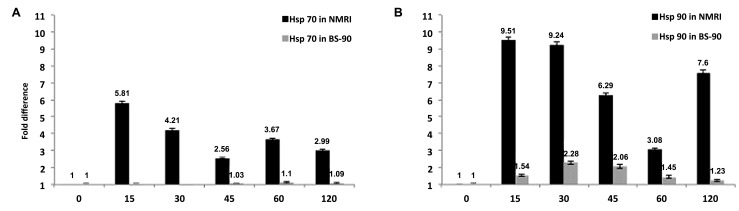
**Temporal modulation of the transcript encoding (A) Hsp 70 or (B) Hsp 90 in susceptible NMRI and resistant (BS–90) *B. glabrata* snail stocks examined by qPCR after exposure (0 – 120 min) to *S. mansoni* miracidia.** Note that expression of the transcripts occurs only in the susceptible but not in the resistant snail (Ittiprasert et al., 2010; Ittiprasert and Knight, 2012).

From studies that compared the temporal regulation in gene expression between resistant and susceptible snails exposed to *S. mansoni*, it was detected that several stress related transcripts were up-regulated early and significantly in susceptible juvenile snails compared to their resistant counterparts (**Figures 1A,B**; Ittiprasert et al., 2010). For example, transcripts encoding heat shock protein (Hsp) 70, Hsp 90, and the reverse transcriptase (RT) domain of the *B. glabrata* non-LTR retro-transposable element, *nimbus*, were detected in susceptible NMRI snails at early time points (5 h) post-exposure compared to the resistant BS-90 snail. Furthermore, upregulation of the aforementioned stress related transcripts was observed in snails exposed to normal but not to irradiated miracidia (Ittiprasert et al., 2010).

## GENOME ORGANISATION IN *B. glabrata* IS SIMILAR TO HIGHER ORGANISMS

**FIGURE 2:**
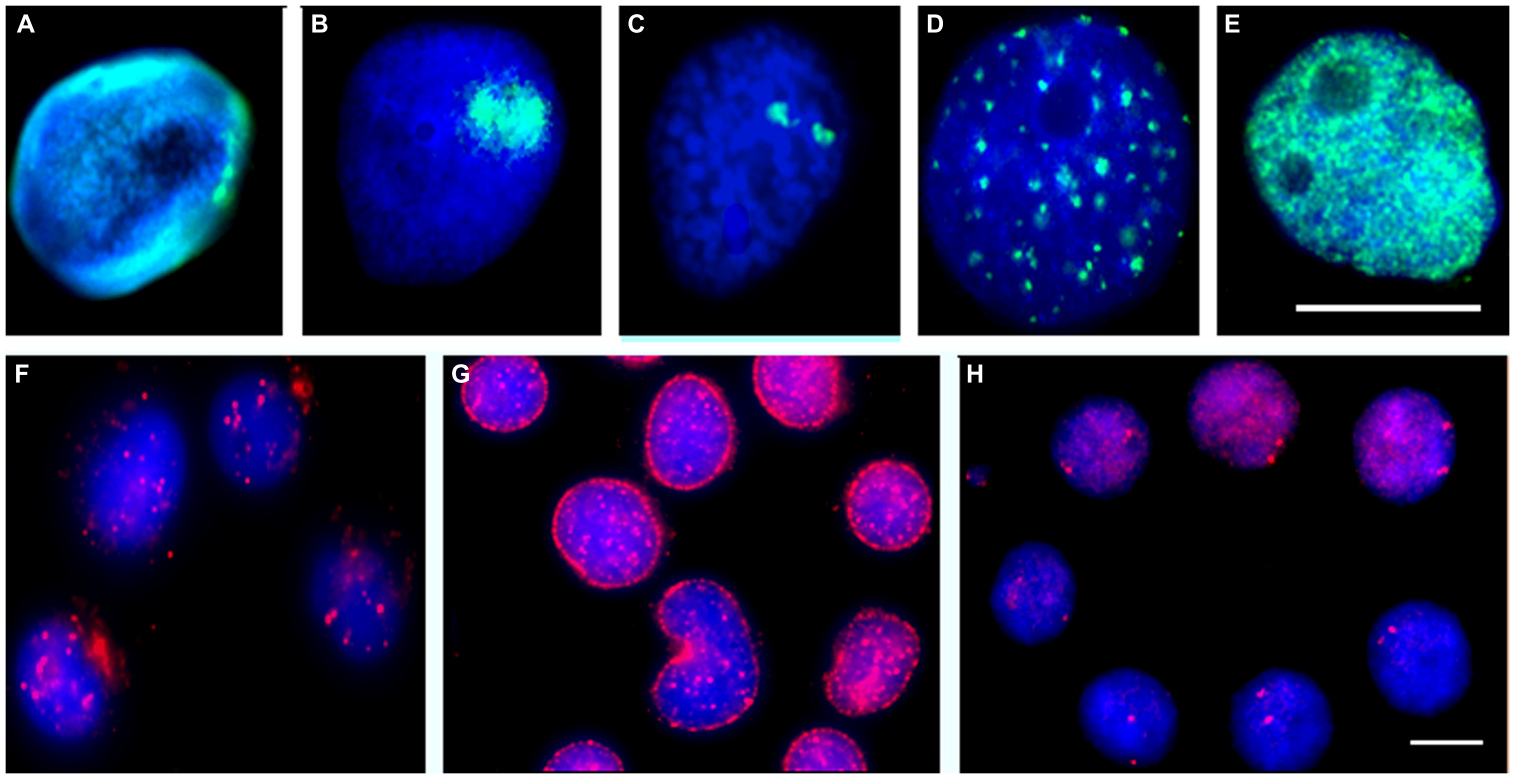
***Biomphalaria glabrata* embryonic (Bge) cells grown *in vitro* or *ex vivo* cells extracted from *B. glabrata* snails stained with antibodies or subjected to FISH.** Antibodies raised against other species were used to reveal similar nuclear structures within snails and Bge cells; these include the nuclear lamina (**A**, green), fibrillarin in the nucleolus (**B**, green), PML bodies (**F**, red) and nuclear myosin (**G**, red). Chromatin modification was revealed using antibodies made against *D. melanogaster* modification that have a broad species range; histon 4 trimethylated K27 (**D**, green) and histone 3 trimethyl K4 (**E**, green). Individual chromosome territories were delineated by allowing 5-bromo-2′-deoxyuridine to be incorporated into replicating DNA and then allowing cells to divide seven times, followed by indirect immunofluorescence with an anti-5-bromo-2′-deoxyuridine antibody (**C**, red). Individual gene loci were revealed by FISH using labeled BAC probes (**H**, red). The DNA of the cells was stained with a DNA intercalating dye 4′6-diamidino-2-phenylindole. Scale bar 10 μm (Drs. Arican-Goktas, 2013 and Odoemelams′, 2009, Ph.D. thesis, Brunel University, UK) (Odoemelam, 2009; Arican-Goktas, 2013).

In order to understand how the parasite is able to induce and even regulate gene expression – such as the heat shock genes, it is imperative to understand the functional genome biology and its organization within the host cell nuclei. Furthermore, to fully appreciate how the parasite is controlling gene expression the structures that are involved in genome organization and behavior also need to be elucidated (see **Figures 2A,B,F,G**).

When originally analyzing the organization of the snail genome within cell nuclei it was hypothesized that it might be more similar to simpler organisms such as *Drosophila melanogaster* and *Caenorhabditis elegans*. Interestingly, it was found that the genome organization within the snail was similar to the mammalian genome. This was discovered by the preparation of a single chromosome painting probe that when used in fluorescence *in situ* hybridization (FISH) delineated individual chromosome territories (**Figure 2C**; Knight et al., 2009), these resembled territories found in higher organisms such as human, mouse (Foster et al., 2005) and pigs (Foster et al., 2012) and was also found to be non-randomly located within cell nuclei. Large and small chromosome territories were also revealed (Odoemelam et al., 2009), although it is as yet not possible to reveal gene density correlation with chromosome location since sequencing of the genome remains unfinished. However, the karyotype of *B. glabrata* has now been completed and several chromosomes have now been named and identified by utilizing bacterial artificial chromosomes (BAC) probes generated by the genome sequencing effort (Arican-Goktas et al., unpublished data). From these data, it is clear that *B. glabrata* chromosomes are not as previously described, i.e., being monomeric since they display differences in size, centromere position, and banding, implying there are gene-rich and gene-poor chromosome regions as well as gene-rich and -poor chromosomes. This advance will be very important for the genome sequencing effort as it will allow genes to be located to a specific chromosome, facilitating assembly, annotation, and organization of the sequence data. The protocol development for mapping onto *B. glabrata* chromosomes by FISH has taken a decade to refine and these methods will be published with the manuscript describing the genome.

The genome has some role in organizing itself within nuclei – through chromatin modification, i.e., the histone code (**Figures 2D,E**), but it also requires many different proteins within nuclei to help position and tether it. A concerted effort has been made to study *B. glabrata* cell nuclei to determine how similar they are to other organisms. As mentioned above, the nucleus of *B. glabrata* appears to be very similar to mammalian cell nuclei (**Figure 2**), containing many nuclear structures that were revealed using indirect immunofluorescence with antibodies reacting with human antigens, such as nuclear lamins (**Figure 2A**), nucleoli (**Figure 2B**), nuclear bodies (**Figure 2F**), nuclear motors (**Figure 2G**), transcription factories and splicing speckles, making this mollusk an interesting model to analyze genome behavior in since it has aspects of both simpler and more complex organisms.

## SCHISTOSOMES INFLUENCE REPOSITIONING OF GENE LOCI IN INTERPHASE NUCLEAR, CORRESPONDING WITH TRANSCRIPTION

The complex relationship between the schistosome parasite and a given compatible species of snail developed over many 1000s of years, and is a co-evolved relationship.

Using labeled BAC probes and FISH analysis, gene loci position has been mapped in *B. glabrata* and found as small foci, very similar to other organisms (**Figure 2H**; Odoemelam et al., 2009). Using a bespoke computer analysis script (Croft, 1999), the positioning of these gene loci is found to be non-random with some genes being found at the nuclear periphery, some in the nuclear interior and some in an intermediate location as for other organisms (Szczerbal et al., 2009; Bourne et al., 2013). However, upon exposure to the parasite either *in vitro* with Bge cells (Knight et al., 2011) or *in vivo* in intact snails we found that specific genes change their nuclear location to a new non-random location correlating with their expression. For most genes attenuated parasite failed to elicit either a relocalization of gene loci, or change in expression. The *in vivo* studies have been most revealing since we were able to employ both susceptible and resistant snails. For gene loci corresponding to Hsp 70, we found overwhelming differences in behavior between the two snail lines. For example, in the susceptible NIMR snail, the gene loci for Hsp 70 moved to a new nuclear location before being expressed whereas no such movement and no gene expression for Hsp 70 were observed in the resistant BS-90 snail, corroborating previously reported differential gene expression data for Hsp 70 between resistant and susceptible snails (Knight et al., 2011). Additionally, when attenuated parasite was used to infect these snails, neither gene movement nor expression of Hsp 70 was observed. From these data, we believe the movement and expression of Hsp 70 is controlled by the parasite infection and may be used to help promote its survival/development in the snail (Arican-Goktas et al., 2014) parasite elicit a gene movement to a new nuclear location for gene expression to occur? We postulate that this requires remodeling of chromatin at specific genome locations and active directed movement to an area of active transcription in the cell nuclei using nuclear motor proteins such as nuclear myosin 1b (**Figure 2G**).

## CONCLUSION

In the 20 years since a molecular approach to decipher mechanisms of the snail host/ schistosome interaction was undertaken – a relationship fashioned over centuries to give each organism a survival advantage over the other, we have discussed in this review how important advances have indeed been made. For example, the long held belief that innate defense molecules of invertebrates, unlike those of vertebrates (e.g., antibody) lack the ability to diversify in their structure has been decisively proven incorrect by the level of diversification revealed in *B. glabrata* FREP transcripts. Likewise, we now also know that schistosomes have evolved a mechanism that is capable of manipulating the genome of a compatible snail host, thereby orchestrating changes in the snail’s cell nuclei that alters gene expression in the parasite’s favor. While these important groundbreaking advances have been made, however, more work remains to be done. For example, we still do not know how schistosomes induce relocalization of gene loci to nuclear compartments for gene expression or silencing. These questions notwithstanding, it is clear that with the entire sequence of the genome soon becoming available, *B. glabrata* should fast become a new model system whereby the effect of a complex pathogen on genome behavior can be examined, enabling us to discover novel pathways that can be interfered with to disable snail transmission of schistosomiasis.

## Conflict of Interest Statement

The authors declare that the research was conducted in the absence of any commercial or financial relationships that could be construed as a potential conflict of interest.
